# Author Correction: The evolution of dam induced river fragmentation in the United States

**DOI:** 10.1038/s41467-024-51659-1

**Published:** 2024-08-21

**Authors:** Rachel A. Spinti, Laura E. Condon, Jun Zhang

**Affiliations:** 1 Hydrology and Atmospheric Sciences, University of Arizona, 1133 James E. Rogers Way, Tucson, AZ 85721 USA; 2 Key Laboratory of VGE of Ministry of Education, Nanjing Normal University, Nanjing, 210000 China

Correction to: *Nature Communications* 10.1038/s41467-023-39194-x, published online 28 June 2023

The original version of this Article contained an error in the description of the results (end of the first paragraph), which incorrectly omitted the following: ‘Cooper et al. ^21^ used the NABD database to calculate 21 different dam impact metrics across the CONUS including river fragmentation and degree of regulation in their analysis of dam impacts on fish assemblages. Our approach is methodologically very similar to theirs (both with respect to metrics and underlaying datasets), but considers evolving impacts over time.’.

The original version of this Article contained an error in the Methods (end of the first paragraph of ‘Datasets’), which incorrectly omitted the following: ‘Roughly ~6,300 dams (roughly 12% of the database and 1.2% of total storage) used in our analysis are missing year of dam construction. For our analysis we assign these dams a construction date of prior to 1920. This assumption can impact our results in some locations as dams with missing construction dates are not evenly distributed. This has a larger impact on fragmentation analysis than degree of regulation as the relative storage fraction of these dams is very small.’.

These have been corrected in the PDF and HTML versions of the Article.

