# Supplementary material for: The evolution of dam induced river fragmentation in the United States
Source: Nat Commun. 2023 Jun 28;14:3820. doi: 10.1038/s41467-023-39194-x (PMC10307825; doi:10.1038/s41467-023-39194-x)
Supplement: Supplementary file 1 — Supplementary Information [file 41467_2023_39194_MOESM1_ESM.pdf]

The Evolution of Dam Induced River Fragmentation in the United States

Examples of the regional differences can be seen by comparing the patterns in the degree of regulation across major river basins. In the Ohio river basin, regulation increased in the headwaters and tributaries over time (Figure 1a and b), but this change was not transmitted to the main stem. The construction of a multitude of dams between 1950 and 2012 is also evident in the increased density of flowlines present in Figure 1b, which is both from medium and large dams. However, the amount of regulation from medium dams only changes along a few reaches in the upper part of the basin (Figure 1b). The middle and lower parts of the main stem of the Ohio maintain the same fraction of regulation from medium dams (40%-60%), despite the addition of dams. Headwaters and tributaries are mostly dominated by medium dams in the Colorado river basin (Figure 1d and e). In 1950, the main stem was mostly regulated by medium dams (Figure 1d). This is still true for the Upper Basin today (Figure 1e), but with the construction of major large dams on the main stem of the Colorado after 1950 it is no longer true for the Lower Basin. In contrast, New England has very few changes in regulation between 1950 and 2012 (Figure 1f and g). Most rivers in this basin are regulated by medium dams, especially in upper reaches. Some of the main stems have less than 40% regulation from medium dams, likely because the drainage area is large enough to support a larger dam.

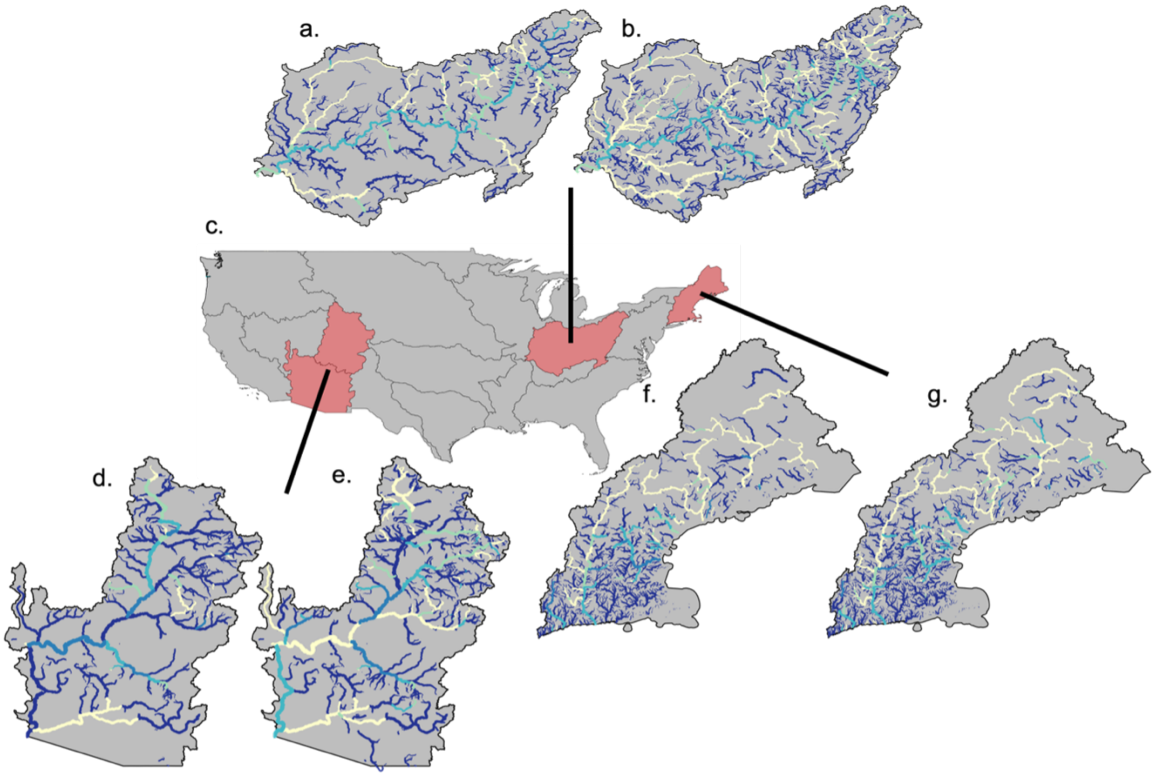

Supplementary Figure 1. Changes in regulation over time vary by basin. Fraction of Degree of Regulation caused by medium dams over time in the Ohio, Colorado, New England basins. *a*. Ohio 1950, *b*. Ohio 2012, *c*. contiguous United States (CONUS) map with highlighted basins, *d*. Colorado 1950, *e*. Colorado 2012, *f*. New England 1950, *g*. New England 2012. The legend is the same as in Figure 3.

Medium dams account for approximately 73% of all fragments nationwide and create the shortest river fragments. While medium dams account for 48% of storage, they are 96% of structures and therefore have a larger effect on river fragmentation than regulation. Fragmentation from medium dams has a similar history to overall dam fragmentation with construction ramping up over time and peaking in the 1980s, but we also observe a pattern of increasing medium dam construction in recent years. Beginning in 1920, medium dams accounted for 40% of the fragments in the US. This percentage has steadily increased over time (Figure 2b), indicating a shift toward medium dam construction. As of 2010, medium dams account for 73% of all fragments nationwide. Fragmentation in the Gulf Coast, Mississippi, North Atlantic, and South Atlantic basins is also dominated by medium dams (fraction of medium dam contribution to fragmentation is greater than 50%).

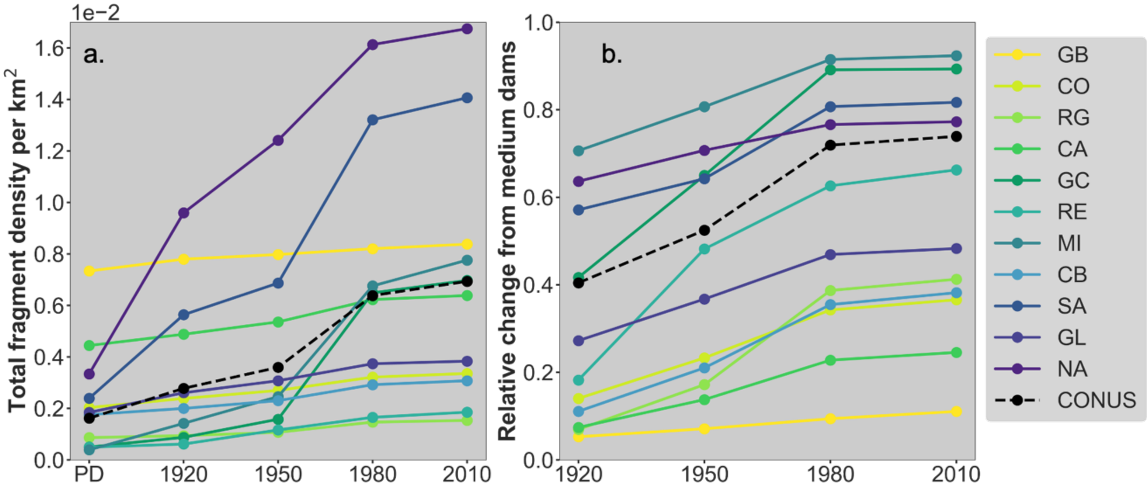

Supplementary Figure 2. Total fragment density has increased over time, partially because of medium dam construction. *a*. Fragment density (the total number of fragments per basin divided by the basin area), where PD is pre-development. *b*. Fraction of fragments caused by medium dams (1-number of fragments from large dams/number of fragments from all dams) over time by major river basin.
